# Manganese in Groundwater in South Asia Needs Attention

**DOI:** 10.1021/acsestwater.2c00442

**Published:** 2022-10-05

**Authors:** M. Feisal Rahman, Muhammad Ashraf Ali, Ahmed I. A. Chowdhury, Peter Ravenscroft

**Affiliations:** † Department of Geography and Environmental Sciences, Northumbria University, Newcastle upon Tyne NE1 8ST, U.K.; ‡ Department of Civil Engineering, Bangladesh University of Engineering and Technology, Ramna, Dhaka 1000, Bangladesh; § Institute of Water and Flood Management, Bangladesh University of Engineering and Technology, Ramna, Dhaka 1000, Bangladesh; ∥ Consultant Hydrogeologist, Girton, Cambridge CB3 0QW, U.K.

The frequent occurrence of manganese (Mn) at elevated concentrations in groundwater adds a new dimension to the already precarious safe water supply scenario in the alluvial plains and deltas of South Asia (SA). An essential micronutrient, Mn may co-occur with iron (Fe) and/or arsenic (As) and can impart a color, odor, or taste to the water at concentrations of >0.02 mg/L.^1^ Adverse effects on neurological development of children from prolonged exposure to Mn in water (∼0.1 mg/L) have been documented^1,2^ (also see Table SI-1). Currently, awareness of Mn among scientists, policy actors, and exposed communities remains low. Despite the growing evidence that Mn in drinking water needs close attention and regular monitoring to avoid excessive intake, in 2011 the World Health Organization (WHO) discontinued the health-based value (HbV) of 0.4 mg/L Mn in drinking water.^3^ Subsequently in 2021, as part of the second addendum to the fourth Guidelines for drinking-water quality (GDWQ), WHO established a new provisional guideline value (pGV) of 0.08 mg/L.^1^ Millions of people in SA are already exposed to Mn above the WHO’s former health-based value (HbV) of 0.4 mg/L. If wells with Mn concentrations above the pGV are considered, then the population exposed to unsafe water would increase significantly.

This Viewpoint provides an overview of the occurrence of Mn in South Asian aquifers and associated health impacts and discusses the implications of discontinuing the former HbV and subsequent introduction of the new pGV. Our recommendations call for greater attention to be paid to Mn in groundwater in SA by scientists and policy makers.

## Occurrence, Mobilization, and Transport of Mn in Groundwater in SA

**Figure 1:**
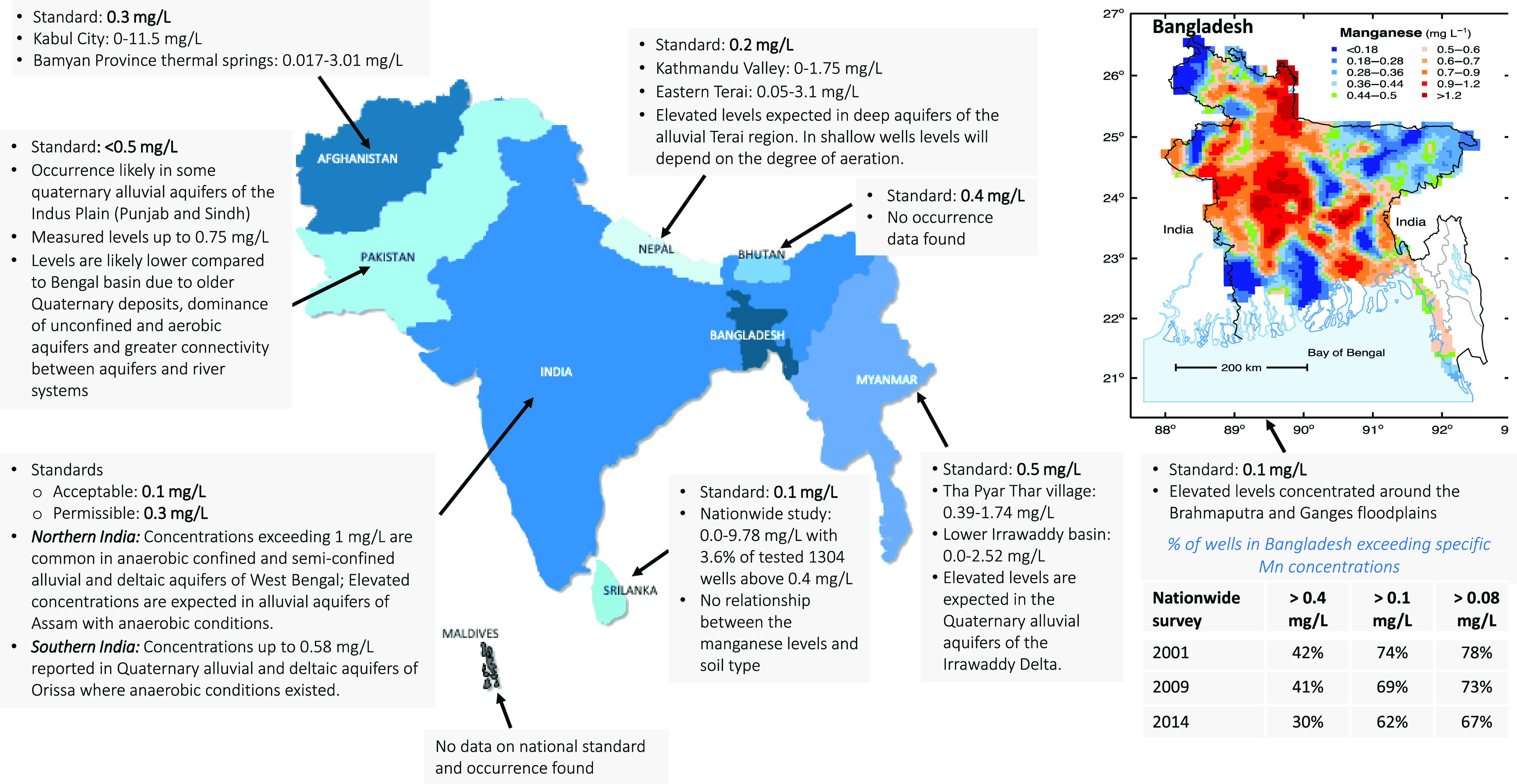
Existing standards and summary of the reported occurrence of Mn in groundwater in SA (see Table SI-2). The inset map reported by the British Geological Survey in 2001 shows the distribution of Mn in Bangladesh.^4^

Mn concentrations frequently exceed 0.4 mg/L in groundwaters in SA (see Figure 1 and Table SI-2). Elevated concentrations have been widely reported in shallow alluvial and deltaic aquifers under reducing conditions. This and high population densities on deltaic and river floodplains have led to a large population in SA being exposed to elevated levels of Mn. For example, in Bangladesh a nationwide survey in 2001 reported 39% of shallow tubewells (<150 m deep) and 2% of deeper wells had >0.4 mg/L Mn and 5% had >2 mg/L with a maximum of 10 mg/L; ∼78% of wells exceeded the WHO pGV of 0.08 mg/L.^4^ A survey in the Indian part of the Bengal Delta reported that 47% of 527 shallow wells and 11% of deep wells exceeded 0.4 mg/L with a maximum of 6 mg/L.^5^ Beyond the Bengal Delta, studies have reported elevated levels of Mn in groundwater in Nepal, Myanmar, Pakistan, Sri Lanka, and Afghanistan (Figure 1). Mn concentrations as high as 25 mg/L have also been detected in several other alluvial and deltaic aquifers, including the Mekong River (Vietnam and Cambodia) and the Red River Delta (Vietnam) in Southeast Asia.^6,7^

In shallow aquifers, mobilization of Mn(II) occurs due to the reduction of Mn(IV) solids present in the subsurface strata to Mn(II). McArthur et al.^5^ reported the reduction of both Mn oxides and Fe oxyhydroxides by microbial metabolism of organic matter/dissolved organic carbon (DOC) in the shallow aquifers of the Bengal Delta. Major sources of DOC include leaching from pit-latrines and sedimentary organic carbon (SOC). These processes form part of a sequence of redox reactions that begin with the reduction of nitrate and mobilization of manganese and proceed *inter alia* to the mobilization of iron and arsenic.^8^ Ying et al.^9^ reported that while arsenic concentrations in groundwater could be predicted well by redox indicators (Eh and dissolved oxygen), Mn shows no significant relationship with either parameter, possibly suggesting the involvement of other parameters and processes in the mobilization of Mn. Ravenscroft et al.^10^ in a survey of deeper aquifers (>150 m) in Bangladesh observed Mn concentrations of >0.4 mg/L in some regions (in the southeast, western, and central regions) having low As levels. Many terrains in SA that are affected by As are also affected by Mn. However, arsenic and manganese are frequently closely juxtaposed but also found to be mutually exclusive when individual wells or aquifer horizons are considered. While deeper aquifers are usually an excellent option for avoiding high arsenic concentrations, care should be taken to determine their suitability regarding Mn and other contaminants and high salinity.^10^

## Health Impacts of Elevated Mn Levels in Water

Elevated levels of Mn in water are concerning as studies have indicated that it is a powerful neurotoxin that may lead to learning disabilities, deficits in intellectual function, compulsive behavior, and attention disorder in children (Table SI-1). For example, Wasserman et al.^2^ observed that exposure to groundwater Mn (mean of 0.8 mg/L) in Araihazar, Bangladesh, was associated with reduction in verbal performance and full-scale IQ score, suggesting a potential neurotoxic impact on children. In the elderly, elevated levels of Mn may cause manganism and Mn-induced Parkinsonism.^1^

In parts of SA, children are at high risk of long-term psychological and social distress because of economic, political, and environmental stressors, and Mn may act as an additional stressor in those situations. Also, there is a lack of knowledge about the potential health impacts of the co-occurrence of Mn and arsenic in SA and other regions.

## Implications of WHO’s Drinking Water Guideline Values for Mn

While concerns over the health impacts of Mn in water were growing, in 2011 WHO revoked the HbV of 0.4 mg/L for Mn in drinking water, suggesting that “this health-based value is well above concentrations of manganese normally found in drinking water”.^3^ However, this rationale appears to have been a major oversight as more than 50 countries globally have reported higher Mn levels in water.^11^ WHO retained the aesthetic guideline value for Mn (0.1 mg/L),^1^ which is lower than the discontinued HbV, yet many people in SA, especially disadvantaged groups, still drink aesthetically unacceptable water, suggesting that an aesthetic threshold is not effective for protecting public health. Therefore, discontinuation of the HbV for Mn has disproportionately affected disadvantaged populations.^11^

Across SA, there is poor awareness of the occurrence and potential health effects of Mn in drinking water despite its frequent detection at elevated levels, and discontinuation of the Mn HbV further undermined the situation. The economic impact of impaired intellectual development on millions of children in SA and around the world could be enormous.

Following calls^11^ to review the decision to discontinue the Mn HbV, WHO recently established a pGV of 0.08 mg/L for total Mn.^1^ Drinking water standards in SA are higher than the proposed pGV (Figure 1). These standards should be re-evaluated given the recent progress on the health impacts of Mn and the pGV. Adopting a standard of 0.08 mg/L Mn would classify a significant fraction of wells in SA unsafe. For example, recent data from Bangladesh suggests at least 20 million people are still exposed to As concentrations of >0.05 mg/L (Bangladesh standard) and roughly double that number are still exposed to As concentrations of >0.01 mg/L (WHO guideline).^12^ If wells with Mn concentrations exceeding 0.08 mg/L are considered, then the population officially exposed to unsafe water would increase significantly. Application of a stringent Mn standard would significantly reduce the coverage of “safe water” in several SA countries, and therefore, it might be very difficult to acquire the required political buy-in. Where the exposed population is very large, countries could adopt a phased implementation of the pGV as a standard, wherein multiple compliance dates are specified sequentially, e.g., a new standard at new public and commercial schemes, retaining the old standard for a limited time at existing schemes, and compliance with the new standard at existing schemes. The exact timing of these dates should be based on national capacities.

Given its toxicity and prevalence, the avoidance and removal of As should remain as the highest priority for SA contexts especially for alluvial and deltaic aquifers. However, the prevalence of elevated Mn concentrations suggests that drinking water treatment systems should also be evaluated for Mn; this would, however, place additional pressure on the limited resources in most countries in SA. For such situations, WHO encouraged “incremental improvements towards meeting the pGV”.^1^ Considering the challenge of achieving the pGV for groundwater, WHO suggests “it is vital that a sufficient supply of microbiologically safe water that is acceptable is always available, even if some guidelines or standards for chemicals such as manganese cannot be immediately met”.^1^

## Options for Removal of Mn from Drinking Water

Avoidance is always the preferred option but this is not always possible. Fortunately, a range of relatively low-cost Mn removal options are available. These include chemical oxidation followed by filtration, adsorption/oxidation on Mn oxide-coated media, softening/ion exchange, and biological filtration. The choice of an appropriate treatment system depends on the detected form (speciation) of Mn in the source water, co-occuring contaminants and the local context (e.g., access and socioeconomic conditions). Several community- and household-level treatment systems have been designed for co-removal of Fe and As. However, such systems often perform poorly for Mn, and there is a need to develop efficient technologies for simultaneous removal of As, Fe, and Mn.^12^

As noted above, deep wells generally have less Mn than shallow wells. However, shifting to deep wells from shallow ones may not always work. Household and rural/small community water treatment systems are usually difficult to maintain. Thus, the widespread, but largely separate, prevalence of both health-impacting As and Mn and the widespread occurrence of objectionable high Fe concentrations can be perceived as an opportunity for public investment in piped water systems (operated by full-time operators and supported by public utilities or their contractors). Such system may offer, especially for rural/small communities, better quantity and quality of water with reduced labor and social conflict, and a higher standard of living and reduced morbidity.

## Concluding Remarks and the Way Forward

To comply with the ethical requirements for groundwater abstraction^10^ pertaining to the occurrence of Mn in groundwater in SA, it is essential (i) to consider the intergenerational impacts of neurological impairment of children (both born and unborn), which provide a strong argument for minimizing exposure, and (ii) to facilitate awareness of the issue to guide rational decision making because devising locally appropriate mitigation strategies may not be possible without informed community participation and feedback. To address knowledge gaps and devise appropriate strategies to minimize exposure, we suggest the following.

- Beyond the Bengal delta, the extent of Mn occurrence in SA is yet to be thoroughly surveyed. Random screening of wells in unsurveyed high-risk areas is a priority (which may be identified using a combination of geology and known water quality parameters). Additionally, concentrations in already identified high-risk areas should be monitored.
- Further research is needed to better understand the mobility and transport mechanisms of Mn in groundwater, its spatial distribution, and its co-occurrence with As.
- Existing water treatment systems may need to be modified or new systems developed for areas with elevated Mn levels. Research should also be undertaken into cost-effective and locally appropriate treatment options for removal of Mn, either alone or where it co-occurs with As, Fe, or both. In these areas, public investment in piped water systems should be considered as a priority option for safe water supply.
- Ecological and longitudinal epidemiological studies (or a combination of both) should be undertaken to ascertain health impacts of prolonged exposure to Mn in water, especially when it coexists with As. In this regard, studies could examine the economic costs of not reducing childhood exposure.
- Finally, given the prevalence of Mn in aquifers in SA, the emerging evidence of health impacts, and WHO’s new pGV, it is essential to raise awareness of policy actors, water suppliers, researchers, and at-risk communities and hence adopt appropriate strategies to minimize exposure to elevated Mn levels and ensure access to safe water for all.

## Supporting Information Available

Effect of exposure to Mn in water on children and occurrence of Mn in SA aquifers (PDF)
