# Supplementary material for: Manganese in Groundwater in South Asia Needs Attention
Source: ACS ES T Water. 2022 Oct 5;3(6):1425–8. doi: 10.1021/acsestwater.2c00442 (PMC10262691; doi:10.1021/acsestwater.2c00442)
Supplement: Supplementary file 1 — ew2c00442_si_001.pdf [file ew2c00442_si_001.pdf]

Supporting Information

MANGANESE IN GROUNDWATER IN SOUTH ASIA NEEDS ATTENTION

M Feisal Rahman[§*], M. Ashraf Ali[‡], Ahmed I. A. Chowdhury[‡‡] and Peter Ravenscroft[Ø]

[§]*Department of Geography and Environmental Sciences, Northumbria University, Newcastle upon Tyne, NE1 8ST, UK.*

[‡]*Department of Civil Engineering, Bangladesh University of Engineering and Technology, Polashi, Ramna, Dhaka-1000, Bangladesh.*

[‡‡]*Institute of Water and Flood Management, Bangladesh University of Engineering and Technology, Ramna, Dhaka-1000, Bangladesh*

[Ø]*Consultant Hydrogeologist, Girton, Cambridge, CB3 0QW, UK*

[*] *Correspondence: mohammad.f.rahman@northumbria.ac.uk*

Table SI-1. Reported neurotoxicity of exposure to Mn in water (MnW) in children (adapted from Rahman et al. 2021).

Location	Reference	Age (years)	Exposure level: Mn in water (MnW)	Key findings/observed impacts
Bangladesh	Wasserman et al., 2006	10	Mean (SD): 795 (± 755) μg/L<br>Range: 4 to 3,908 μg/L	MnW was associated with reduced full-scale performance, and verbal raw score, in a dose-response manner suggesting potential neurotoxic impact of Mn exposure on children.
Bangladesh	Khan et al., 2011	8-11	Median: 650 μg/L<br>Mean (SD): 889 (±784) μg/L<br>Range: 40–3443 μg/L	Increased MnW exposure was found to be associated with problematic behaviour including both externalising and internalising behaviour with significantly more strongly related to the former.
Bangladesh	Khan et al., 2012	8-11	Median: 1301.6 μg/L<br>Mean (SD): 1388 (±866) μg/L<br>Range: 10-5710 μg/L	Exposure to high Mn concentrations >400 μg/L may affect children's math performance.
Bangladesh	Rahman et al., 2017	10	Median concentrations:<br>Prenatal: 204 μg/L;<br>At age 5: 228 μg/L;<br>At age 10: 339 μg/L	Elevated prenatal and early childhood exposure to MnW appeared to increase the risk of children's behavioural problems at 10 y of age (e.g., increased risk of conduct problems particularly in boys; decreased risk of emotional problems and low prosocial scores for prosocial behaviour in girls). Elevated prenatal MnW exposure was positively associated with cognitive function in girls, whereas boys appeared to be unaffected.
Canada	Bouchard et al., 2018	6-13	Geometric mean: 6.3 μg/L;<br>Arithmetic mean was 62 μg/L	Exposure levels were relatively low during this study. No clear association between exposure to Mn and cognitive development in this sample of school-age

Location	Reference	Age (years)	Exposure level: Mn in water (MnW)	Key findings/observed impacts
			48% of children consuming water <5 μg/L, 25% >50 μg/L, and 4% >400 μg/L;	children were observed. However, data indicated there might sex-disaggregated associations.
Canada	Bouchard et al., 2011	6-13	Median value: 34 μg/L (range 1-2700 μg/L)	Exposure at common levels in groundwater is associated with intellectual impairment in children.
Canada	Bouchard et al., 2007		Two wells with mean concentrations at 610 μg/L and mean 160 μg/L	Exposure to high level of Mn was reported to be linked to increased hyperactive and oppositional behaviors in the classroom.
Canada	Kullar et al. 2019	5.9-13.7 (sample pooled from Bouchard et al. 2011 and 2018)	Geometric mean: 12.3 μg/L<br>Arithmetic mean: 84.8 μg/L<br>Range: 0.01 μg/L to 2701 μg/L	Estimated drinking water Mn benchmark concentrations for a 1%, 2% and 5% decrease of performance IQ in boys were 185, 375 and 935 μg/L and 78, 95, 192 μg/L for girls.
Denmark	Schullehner et al. 2020	Longitudinal exposure during the first 5 years of life for children born between 1992-2007	Five exposure categories:<br><5 μg /L, >19–34 μg/L, >34–100, >100 μg/L	Increasing levels of Mn in drinking water was associated with an increased risk of attention-deficit hyperactivity disorder (ADHD)-Inattentive subtype. Associations between Mn and ADHD were more pronounced in females than in males when exposure was based on the highest level in drinking water, whereas associations were similar for females and males when based on time-weighted average exposures. Findings suggest a need for a formal health-based drinking water guideline value for Mn.
North America;	Oulhote et al., 2014		Geometric mean: 20 μg/L	Exposure to manganese in water was associated with poorer neurobehavioral performances in children, even at low levels commonly encountered in North America,

Location	Reference	Age (years)	Exposure level: Mn in water (MnW)	Key findings/observed impacts
multiple locations				with a steeper decline in performance at MnW >100 μg/L. Higher levels of exposure to Mn are associated with poorer performance of memory, attention, and motor functions, but not hyperactivity, in children.
Greece	Kondakis et al. 1988	≥ 50	Range: 3.6-2300 μg/L	Prevalence of neurological signs of chronic Mn poisoning associated with progressive increases in Mn levels in drinking water.

Table SI-2. Summary of Occurrence of Mn in water in South Asian Aquifers.

Country	Mn national standard values; Occurrence data and other relevant info
Afghanistan	• Standard: 0.3 mg/L<br>• Kabul City: 0-11.5 mg/L (mean 0.74) (Gesim and Okazaki 2018)<br>• Kabul City: 0.43-2.65 mg/L (Wafa et al. 2020)<br>• Bamyan Province thermal springs: 0.017-3.01 mg/L (Jawadi et al. 2021)
Bangladesh	• Standard: 0.1 mg/L<br>• A nationwide survey in 2001 of wells showed concentrations ranging from 0.001-9.98 mg/L with 42% wells > 0.4 mg/L, 73% wells > 0.1 mg/L and 78% well > 0.08 mg/L (BGS-DPHE 2001)<br>• A follow up survey in 2009 of wells showed concentrations ranging from 0.001-9.1 mg/L with 42% wells > 0.4 mg/L, 73% wells > 0.1 mg/L and 78% well > 0.08 mg/L (BBS/UNICEF 2011)<br>• Another survey in 2014 of wells showed concentrations ranging from 0.01-8.71 mg/L with 42% wells > 0.4 mg/L, 73% wells > 0.1 mg/L and 78% well > 0.08 mg/L (Ravenscroft et al. 2014)<br>• Elevated levels are concentrated around the Brahmaputra and Ganges flood plains (BGS-DPHE 2001, Smedley 2003)
Bhutan	• Standard: 0.4 mg/L<br>• No occurrence data available
India	• Standard:<br>o Acceptable: 0.1 mg/L<br>o Permissible: 0.3 mg/L<br>• *Northern India:* Concentrations exceeding 1 mg/L are common in anaerobic confined and semi-confined alluvial and deltaic aquifers of West Bengal; Elevated concentrations are expected in alluvial aquifers of Assam with anaerobic conditions (Smedley 2004)<br>• *Southern India:* Concentrations up to 0.58 mg/L reported in quaternary alluvial and deltaic aquifers of Orissa where anaerobic conditions existed (Smedley 2000)
Maldives	No information available
Myanmar*	• Standard: 0.5 mg/L<br>• Tha Pyar Thar village: 0.39-1.74 mg/L (Bacquart et al. 2015)<br>• Lower Irrawaddy basin: 0.0-2.52 mg/L (median 0.25 mg/L) (van Geen et al. 2014)<br>• Elevated levels are expected in the quaternary alluvial aquifers of the Irrawaddy Delta (Smedley 2020)
Nepal	• Standard: 0.2 mg/L<br>• Kathmandu Valley: 0-1.75 mg/L (median 0.34) (Shrestha et al. 2016)<br>• Eastern Terai: 0.05-3.1 mg/L (median 0.21 mg/L) (Mahato et al. 2018)<br>• Elevated levels are expected in deep aquifers of the alluvial Terai region. In shallow wells levels will depend on the degree of aeration (Smedley 2001a)
Pakistan	• Standard: < 0.5 mg/L

	• Occurrence likely in some quaternary alluvial aquifers of the Indus Plain (e.g. Punjab and Sindh Province) (Smedley 2001)<br>• Measured levels up to 0.75 mg/L (Smedley 2001)<br>• Punjab: 0.004-0.8 mg/L (median 0.18 mg/L) (Shakoor et al. 2015)<br>• In the field area in southern Sindh, concentrations of Mn in groundwater exceed 0.4 mg/L in 11% of groundwaters, with a maximum of 0.7 mg/L, as a result of reduction of sedimentary manganese oxides (Naseem and McArthur 2018)<br>• Levels are likely lower compared to Bengal basin due to older Quaternary deposits, dominance of unconfined and aerobic aquifers and greater connectivity of aquifers with river systems (Smedley 2001)
Sri Lanka	• Standard: 0.1 mg/L<br>• Nationwide study: 0.0-9.78 mg/L with 3.6% of tested 1304 wells above 0.4 mg/L. (Herath et al. 2017)<br>• No relationship between the manganese levels and soil type (Herath et al. 2017)

REFERENCES

Bacquart, T., Frisbie, S., Mitchell, E., Grigg, L., Cole, C., Small, C. and Sarkar, B. 2015. Multiple inorganic toxic substances contaminating the groundwater of Myingyan Township, Myanmar: Arsenic, manganese, fluoride, iron, and uranium. Sci. Total Environ., 517, pp.232-245.

BBS/UNICEF. 2011. Bangladesh National Drinking Water Quality Survey 2009. Bangladesh Bureau of Statistics and UNICEF, Dhaka.

Bouchard, M., Laforest, F., Vandelac, L., Bellinger, D., & Mergler, D. 2007. Hair manganese and hyperactive behaviors: pilot study of school-age children exposed through tap water. Environ. Health Persp. 115(1), 122-127. https://doi.org/10.1289/ehp.9504

Bouchard, M.F., Sauvé, S., Barbeau, B., Legrand, M., Brodeur, M.È., Bouffard, T., Limoges, E., Bellinger, D.C. & Mergler, D. 2011. Intellectual impairment in school-age children exposed to manganese from drinking water. Environ. Health Persp. 119(1), 138-143. https://doi.org/10.1289/ehp.1002321

Bouchard, M. F., Surette, C., Cormier, P., & Foucher, D. 2018. Low level exposure to manganese from drinking water and cognition in school-age children. Neurotoxicology 64, 110-117. https://doi.org/10.1016/j.neuro.2017.07.024

British Geological Survey (BGS)/Government of Bangladesh Department of Public Health Engineering (DPHE). 2001. Arsenic contamination of groundwater in Bangladesh. BGS technical report, WC/00/19. https://nora.nerc.ac.uk/id/eprint/11986/ (Accessed 30 August 2022).

Herath, H.A.S., Kubota, K., Kawakami, T., Nagasawa, S., Motoyama, A., Weragoda, S.K., Chaminda, G.T. and Yatigammana, S.K., 2017. Potential risk of drinking water to human health in Sri Lanka. Environ. Forensics, 18(3), pp.241-250.

Houben, G., Tünnermeier, T., Eqrar, N. and Himmelsbach, T., 2009. Hydrogeology of the Kabul Basin (Afghanistan), part II: groundwater geochemistry. Hydrogeology journal, 17(4), pp.935-948.

Jawadi, H.A., Malistani, H.A., Moheghy, M.A. and Sagin, J., 2021. Essential trace elements and arsenic in thermal springs, Afghanistan. Water, 13(2), p.134.

Khan, K., Factor-Litvak, P., Wasserman, G.A., Liu, X., Ahmed, E., Parvez, F., Slavkovich, V., Levy, D., Mey, J., van Geen, A. & Graziano, J.H. (2011). Manganese exposure from drinking water and children's classroom behavior in Bangladesh. Environ. Health Persp. 119(10), 1501-1506. https://doi.org/10.1289/ehp.1003397

Khan, K., Wasserman, G.A., Liu, X., Ahmed, E., Parvez, F., Slavkovich, V., Levy, D., Mey, J., van Geen, A., Graziano, J.H. & Factor-Litvak, P. (2012). Manganese exposure from drinking water and children's academic achievement. Neurotoxicol., 33(1), 91-97. https://doi.org/10.1016/j.neuro.2011.12.002

Kondakis, X. G., Makris, N., Leotsinidis, M., Prinou, M., & Papapetropoulos, T. (1989). Possible health effects of high manganese concentration in drinking water. Arch. Environ. Heal. 44(3), 175-178. https://doi.org/10.1080/00039896.1989.9935883

Kullar, S.S., Shao, K., Surette, C., Foucher, D., Mergler, D., Cormier, P., Bellinger, D.C., Barbeau, B., Sauvé, S. & Bouchard, M.F. (2019). A benchmark concentration analysis for manganese in drinking water and IQ deficits in children. Environ. Intl., 130, p.104889. https://doi.org/10.1016/j.envint.2019.05.083

Mahato, S., Mahato, A., Karna, P.K. and Balmiki, N., 2018. Investigating aquifer contamination and groundwater quality in eastern Terai region of Nepal. BMC Res. Notes, 11(1), pp.1-7.

Naseem, S. and McArthur, J.M., 2018. Arsenic and other water-quality issues affecting groundwater, I ndus alluvial plain, Pakistan. Hydrol. Proces., 32(9), pp.1235-1253.

Oulhote, Y., Mergler, D., Barbeau, B., Bellinger, D.C., Bouffard, T., Brodeur, M.È., Saint-Amour, D., Legrand, M., Sauvé, S. & Bouchard, M.F. (2014). Neurobehavioral function in school-age children exposed to manganese in drinking water. Environ. Health Persp. 122(12), 1343-1350. https://doi.org/10.1289/ehp.1307918

Rahman, M.F., Mahmud, M.J., Sadmani, A.A., Chowdhury, A.I., Anderson, W.B., Bodruzzaman, A.B. and Huq, S., 2021. Previously unrecognized potential threat to children from manganese in groundwater in rohingya refugee camps in Cox's Bazar, Bangladesh. Chemosphere, 266, p.129128.

Rahman, S. M., Kippler, M., Tofail, F., Bölte, S., Derakhshani Hamadani, J., & Vahter, M. (2017). Manganese in drinking water and cognitive abilities and behavior at 10 years of age: a prospective cohort study. Environ. Health Persp. 125(5), 057003. https://doi.org/10.1289/EHP631

Ravenscroft, P., Kabir, A., Ibn Hakim, S.A., Ibrahim, A.K.M., Ghosh, S.K., Rahman, M.S., Akhter, F. and Sattar, M.A., 2014. Effectiveness of public rural waterpoints in Bangladesh with special reference to arsenic mitigation. Journal of Water, Sanitation and Hygiene for Development, 4(4), pp.545-562.

Schullehner, J., Thygesen, M., Kristiansen, S.M., Hansen, B., Pedersen, C.B. & Dalsgaard, S. (2020). Exposure to manganese in drinking water during childhood and association with attention-deficit hyperactivity disorder: A nationwide cohort study. Environ. Health Persp., 128(9), p.097004. https://doi.org/10.1289/EHP6391

Shakoor, M.B., Niazi, N.K., Bibi, I., Rahman, M.M., Naidu, R., Dong, Z., Shahid, M. and Arshad, M., 2015. Unraveling health risk and speciation of arsenic from groundwater in rural areas of Punjab, Pakistan. Intl. J. Environme. Res. Pub. Health, 12(10), pp.12371-12390.

Smedley, Pauline. 2000. Groundwater quality: Southern India. British Geological Survey. https://nora.nerc.ac.uk/id/eprint/516323/ (accessed: 30 August 2022).

Smedley, P. 2001a. Groundwater quality: Nepal. British Geological Survey. https://nora.nerc.ac.uk/id/eprint/516319/ (accessed: 30 August 2022).

Smedley, P. 2001b. Groundwater quality: Pakistan. British Geological Survey. https://nora.nerc.ac.uk/id/eprint/516322/ (accessed: 30 August 2022).

Smedley, P. 2003. Water quality fact sheet: Manganese. British Geological Survey. https://nora.nerc.ac.uk/id/eprint/516303/ (accessed: 30 August 2022).

Smedley, P. 2004. Groundwater quality: Northern India. British Geological Survey. https://nora.nerc.ac.uk/id/eprint/516321/ (accessed: 30 August 2022).

Smedley, P. 2020. Groundwater quality: Myanmar. British Geological Survey. https://nora.nerc.ac.uk/id/eprint/527955/ (accessed: 30 August 2022).

van Geen, A., Win, K.H., Zaw, T., Naing, W., Mey, J.L. and Mailloux, B., 2014. Confirmation of elevated arsenic levels in groundwater of Myanmar. Sci. Total Environ., 478, pp.21-24.

Wafa, W., Hairan, M.H. and Waizy, H., 2020. The impacts of urbanization on Kabul City's groundwater quality. Int. J. Adv. Sci. Technol, 29(4), pp.10796-10809.

Wasserman, G.A., Liu, X., Parvez, F., Ahsan, H., Levy, D., Factor-Litvak, P., Kline, J., van Geen, A., Slavkovich, V., LoIacono, N.J. and Cheng, Z. (2006). Water manganese exposure and children's intellectual function in Araihazar, Bangladesh. Environ. Health Persp. 114(1), 124-129. https://doi.org/10.1289/ehp.8030
